# Does culture-tourism integration policy enhance urban ecological resilience? Evidence from a multi-period difference-in-differences analysis of Chinese cities

**DOI:** 10.3389/fpubh.2026.1870242

**Published:** 2026-07-28

**Authors:** Fei Lu, Jinsu Wang, Xinglong Zhai

**Affiliations:** 1 College of Literature and History, Weifang University, Weifang, China; 2 College of Education, Weifang University, Weifang, China

**Keywords:** culture-tourism integration, difference-in-differences, double machine learning, ecological resilience, environmental health, green technology innovation, industrial structure upgrading, public environmental awareness

## Abstract

**Objective:**

Global climate change and intensive human activities have posed escalating threats to urban ecosystem stability, underlining the critical role of strengthening ecological resilience in safeguarding environmental health and sustainable urban development. However, whether comprehensive industrial policies such as culture-tourism integration can effectively enhance ecological resilience remains insufficiently examined, particularly through causal inference approaches.

**Methods:**

Using panel data from 284 prefecture-level cities in China spanning 2010–2023, this study employs a multi-period difference-in-differences design as the core identification strategy to investigate the impact of culture-tourism integration policies on urban ecological resilience. A battery of robustness checks including PSM-DID, placebo tests, instrumental variable estimation, and double machine learning are adopted to support the reliability and plausibility of the results.

**Results:**

The results demonstrate that these policies significantly enhance urban ecological resilience, and this conclusion remains robust across multiple specifications. Mechanism analysis reveals that culture-tourism integration policies operate via three potential transmission channels: industrial structure upgrading, green technology innovation, and increased public environmental awareness. Heterogeneity analysis further reveals that the policy effects are statistically significant across all regions but exhibit greater marginal effects in central and western regions; the effects are more pronounced in non-designated historical and cultural cities and in higher-tier cities such as municipalities and provincial capitals.

**Conclusion:**

These findings provide robust empirical evidence that integrated culture-tourism strategies can synergize economic growth and ecological conservation, may generate public health co-benefits and have potential implications for environmental health. This study yields important policy implications for environmental health governance and sustainable development in emerging economies in alignment with the United Nations Sustainable Development Goals.

## 1 Introduction

Against the backdrop of dual disturbances from global climate change and high-intensity human activities, ecosystem degradation has become a universal challenge restricting global sustainable development (1). In this context, ecological resilience—defined as the core capacity of a system to resist shocks, absorb disturbances, and achieve adaptive evolution (2)—has evolved from a peripheral concept to a core paradigm for global environmental governance. However, the traditional “rigid protection” model often encounters governance bottlenecks due to its neglect of local development demands, making it particularly urgent to explore flexible paths for the synergy of protection and development (3). In this regard, culture-tourism integration demonstrates unique theoretical potential. It is not a mere industrial superposition, but a process that redefines human-environment interactions at the micro level through the ecological reconstruction of spatial functions and the realization of ecological product value. Theoretically, this process can effectively alleviate the pressure of high-consumption economic activities on ecosystems, and relies on the agglomeration of green factors and the strengthening of environmental regulation to provide an inherent driving force for the improvement of ecological resilience. As a typical representative in this field, China has been exploring local practices of culture-tourism integration since 2009. In particular, the national institutional restructuring in 2018 elevated it to a top-level design, prompting various cities to issue specialized policies intensively, thus forming a large-scale quasi-natural experiment. Yet a practical paradox has emerged in the rapid advancement of these policies: some regions have fallen into a vicious cycle where “the more excessive the development, the more fragile the ecology” due to the extensive exploitation of resources and landscape homogenization. This gives rise to two critical research questions: can the substantive implementation of culture-tourism integration policies effectively enhance urban ecological resilience, and what are their underlying transmission paths and boundary effects? An in-depth analysis of these issues will not only help address ecosystem degradation, but also may have potential implications for reducing environmental exposure risks, thereby benefiting environmental health and providing empirical evidence for emerging economies to advance ecological governance through industrial policies.

Existing research on the effects of culture-tourism integration has primarily focused on the economic dimension, confirming that it can significantly strengthen regional economic resilience through paths such as consumption expansion and industrial coordination (4). However, attention to the ecological dimension remains relatively insufficient. At present, discussions on the ecological effects of culture-tourism development are still marked by a dual divergence between “ecological damage” and “ecological protection” (5). A small number of cutting-edge studies have begun to move beyond the traditional development perspective and explore the synergistic relationship between culture-tourism integration and ecological resilience. For example, Feng et al. found that culture-tourism integration acts as the order parameter dominating the evolution of ecological resilience in the Yellow River Basin (6). Nevertheless, such studies mostly rely on static evaluation methods such as the coupling coordination degree model, which can hardly avoid endogeneity bias and fail to effectively identify the net effect of culture-tourism integration on ecological resilience improvement and its micro-level transmission mechanisms.

In fact, clarifying the ecological empowerment effect of culture-tourism integration requires moving beyond a single industrial perspective and examining it within the macro framework of the driving logic of ecological resilience. Existing studies indicate that, in addition to natural background factors such as climate fluctuations and topography (7), human interventions represented by policy shocks have been widely proven to be critical driving forces for enhancing ecological resilience. In recent years, the academic community has intensively evaluated specialized policies such as low-carbon city pilots and sponge city construction, confirming that these policies can significantly improve ecological resilience through pathways including green technology innovation and infrastructure improvement (8–10). However, as a comprehensive industrial policy promoted systematically top-down that profoundly reshapes the human-environment spatial relationship, specialized culture-tourism integration policies have not yet been incorporated into the policy evaluation toolkit for ecological resilience, revealing an obvious “policy evaluation blind spot.”

In summary, the existing literature has achieved fruitful results regarding the economic effects of culture-tourism integration and the traditional driving factors of ecological resilience, providing a solid theoretical foundation for this study. Nevertheless, relevant research can be further advanced in the following aspects. In terms of causal inference, most existing studies on the ecological effects of culture-tourism are limited to correlation analysis or static synergy measurement, which cannot effectively eliminate endogeneity bias caused by complex economic and social environments. Consequently, the net effect of culture-tourism integration on ecological resilience improvement still lacks rigorous micro-level evidence. From the perspective of policy instruments, previous policy evaluations of ecological governance have mostly focused on command-and-control special pilots, leaving industry-collaborative policies—such as culture-tourism integration, which features both economic incentives and spatial restructuring functions—largely absent from the analytical framework of ecological resilience. Furthermore, regarding transmission mechanisms and contextual constraints, the systematic examination of how culture-tourism integration crosses industrial boundaries and achieves ecological empowerment through multi-dimensional pathways such as economic restructuring, technological progress, and social governance remains insufficient. Meanwhile, the heterogeneous performance of such effects across cities with different resource endowments has yet to be precisely identified. Addressing these research gaps, this study regards the culture-tourism integration policy as a quasi-natural experiment. Using panel data of Chinese prefecture-level cities and adopting a multi-period difference-in-differences model, this paper aims to scientifically evaluate the causal effect of culture-tourism integration policies on urban ecological resilience, and systematically decompose its underlying mechanisms and heterogeneous characteristics.

The potential marginal contributions of this paper are threefold: First, this study expands the research perspective and causal identification strategy. To the best of our knowledge, this is the first study to identify the causal effect of culture-tourism integration policy on urban ecological resilience. It breaks through the limitation of “emphasizing economy over ecology” in existing effect evaluations, and systematically incorporates culture-tourism integration policies into the analytical framework of ecological resilience. Based on a quasi-natural experiment, this paper accurately identifies the net policy effect and effectively mitigates endogeneity disturbances caused by reverse causality and omitted variables, thus providing rigorous micro-evidence for understanding the green dividend of culture-tourism integration. Second, this study deeply deconstructs the micro-level transmission mechanisms. We reveal three novel and distinctive impact mechanisms: industrial structure upgrading, green technology innovation, and public environmental awareness. From the three dimensions of industrial structure upgrading, green technology innovation, and public environmental awareness, this paper systematically analyzes the multiple pathways through which culture-tourism integration policies empower ecological resilience. This not only overcomes the constraints of single-dimensional explanations in previous studies but also reveals the internal logic that culture-tourism integration drives ecosystem restoration via the synergistic linkage of “economic structure optimization–technical support enhancement–social co-governance participation,” thereby extending the research boundaries of the environmental effects of culture-tourism integration. Third, this study precisely depicts the policy heterogeneity and dynamic characteristics. We provide new empirical evidence linking industrial policy to environmental health, which strongly supports the achievement of SDG 3 (good health), SDG 11 (sustainable cities), and SDG 13 (climate action). It fully examines the differentiated ecological effects of culture-tourism integration policies in cities with different resource endowments and development stages, and identifies the boundary conditions of policy implementation. These findings not only offer localized implications for the precise implementation and dynamic optimization of culture-tourism integration policies with Chinese characteristics, but also provide a Chinese experience for global emerging economies to resolve the dual paradox of “protection versus development” and advance the United Nations SDGs through industry-collaborative policies.

## 2 Policy background and research hypotheses

### 2.1 Policy background

Promoting the integrated development of culture and tourism represents a major strategic initiative of China in response to high-quality economic development and the pursuit of a better life for its people. Since 2009, when the former Ministry of Culture and the National Tourism Administration jointly issued the *Guiding Opinions on Promoting the Integrated Development of Culture and Tourism*, local governments have launched independent explorations into the localized practice of culture-tourism integration. The official establishment of the Ministry of Culture and Tourism in 2018 elevated this initiative to the level of national top-level design, initiating systematic and in-depth institutional restructuring and prompting prefecture-level cities to intensively introduce targeted policies. Notably, culture-tourism integration policies in the new era are no longer confined to industrial superposition at the economic level. Instead, they have deeply incorporated the ecological civilization vision that “clear waters and green mountains are invaluable assets.” These policies emphasize strict adherence to ecological redlines, advocate green and low-carbon development, and promote the realization of the value of ecological products in the development of cultural and tourism resources. Such institutional arrangements reshape urban industrial spaces while profoundly transforming regional resource utilization patterns and environmental governance models, exerting important impacts on the pressure-state and response mechanisms of urban ecosystems. This provides a solid realistic foundation for this study to examine the effects of culture-tourism integration policies on urban ecological resilience.

This study employs panel data from 284 prefecture-level cities in China covering the period 2010–2023, among which 243 cities have successively issued specialized policies for culture-tourism integration. The validity of treating this policy as a quasi-natural experiment is grounded in the following logic: First, the sample is broadly representative and avoids selection bias. An implementation rate of 85.56% ensures that the sample is nationally representative, while the remaining cities that have not introduced such policies can serve as an effective control group. Second, the policy is implemented staggeredly, satisfying the prerequisites of the multi-period difference-in-differences model. The policy is not rolled out in a “one-size-fits-all” manner; instead, it has been gradually implemented year by year. This provides rich variation in treatment timing for the econometric model and allows for the effective identification of the net policy effect. Third, the policy is driven by top-down mandates and exhibits strong exogeneity. The introduction of the policy is mainly motivated by national top-level design and local resource endowments, rather than directly determined by the current level of urban ecological resilience, thus effectively mitigating reverse causality. Fourth, the policy breaks administrative barriers and generates a profound institutional shock. Rather than being a simple industrial support measure, this policy entails institutional restructuring in resource coordination, spatial planning, and other fields. It imposes a substantial institutional impact on urban environmental governance and thus possesses sufficient strength as the core explanatory variable.

### 2.2 Theoretical analysis and research hypotheses

Culture-tourism integration represents a critical pathway for cities to transform from traditional sightseeing-oriented growth toward high-quality and sustainable development. Its core lies in addressing the negative environmental externalities arising from market failure in conventional tourism development (5, 11). From the perspective of policy instrument theory, culture-tourism integration policies are not simply industrial incentive tools; instead, they form an institutional system of “coordinated protection and development” in urban spaces through the synergistic deployment of environmental, supply-side, and demand-side policy instruments (12). On the one hand, culture-tourism integration policies internalize ecological costs via environmental instruments. Measures including green finance and cultural tourism consumption pilots effectively foster the sustainable development of the tourism economy (13) and strengthen the capacity for dynamic environmental monitoring and risk early warning (14). On the other hand, the policy consolidates the ecological support base through supply-side instruments. Digital technology empowerment significantly promotes the high-quality development of the culture-tourism industry and improves the efficiency of information processing and resource allocation in ecosystems (15, 16). In addition, culture-tourism integration policies guide green consumption behaviors through demand-side instruments, narrow the urban–rural income gap, improve resident wellbeing (17, 18), and unlock the value realization channels of ecological products. In doing so, a virtuous cycle of “tourism promoting culture, and culture nurturing ecology” takes shape. Accordingly, we propose the following hypothesis.

*Hypothesis 1*: Culture-tourism integration policies can significantly improve urban ecological resilience.

The ecological transformation of industrial structure serves as a key buffering mechanism for cities to resist external shocks and maintain system stability. Industrial structure upgrading drives the economic system toward a green, low-carbon transition characterized by high added value, low energy consumption, and low pollution, which reduces disturbances from industrial activities to the ecological environment at the source and enhances the resistance and resilience of urban ecosystems (17, 19, 20). Meanwhile, industrial structure optimization helps improve resource allocation efficiency and technological innovation capacity, providing technical support and material guarantees for ecological risk prevention and environmental governance, thereby enhancing the adaptability of ecosystems (21–24). As important industrial guidance policy, culture-tourism integration policies promote the agglomeration of capital, technology, talent, and other factors toward modern service industries such as cultural tourism, creative design, and digital content through factor reallocation. This drives the industrial system to transform from the traditional high-consumption model to a green and innovative model. Existing studies confirm that policies such as smart city initiatives and low-carbon pilot programs can significantly improve urban ecological resilience through the mediating path of industrial structure upgrading (25, 26). Such structural transformation not only directly reduces ecological pressure in the production process but also drives the green upgrading of upstream and downstream industries through industrial linkages and technology spillovers, thereby systematically enhancing urban ecological resilience (27, 28). Accordingly, we propose the following hypothesis.

*Hypothesis 2*: Culture-tourism integration policies improve urban ecological resilience by promoting industrial structure upgrading.

Green technology innovation constitutes the core endogenous driver for ecosystems to achieve self-renewal and precise risk governance. By advancing clean energy substitution, energy conservation, emission reduction, and pollution control technologies, green technology innovation reduces disruptions to the ecological environment from economic activities at the source, thereby strengthening the resistance and resilience of urban ecosystems (8, 21). Meanwhile, green technologies can improve resource allocation efficiency and provide technical support for the accurate identification, dynamic monitoring, and intelligent governance of ecological risks, further enhancing the adaptability and learning capacity of the system (23, 29). Different from traditional command-and-control regulation, culture-tourism integration policies establish an induced institutional environment that provides clear market signals and incentive-compatible mechanisms for the research, development, and application of green technologies. By setting green project standards, offering R&D subsidies, and creating green consumption scenarios, these policies effectively reduce the transaction costs of green innovation and motivate enterprises to adopt clean technologies and digital governance tools (30, 31). Such a policy-driven technological paradigm shift not only lowers the ecological footprint of cultural and tourism activities at the source but also significantly improves the learning and adaptive capacities of urban ecosystems through digital monitoring, intelligent early warning, and other means (17). Accordingly, we propose the following hypothesis.

*Hypothesis 3*: Culture-tourism integration policies enhance urban ecological resilience by promoting green technology innovation.

The improvement of urban ecological resilience depends not only on hardware technologies and infrastructure but also on a soft social cognitive foundation. As an informal institutional constraint, public environmental awareness can generate social supervision and public pressure, pushing local governments to strengthen environmental regulation and increase investment in ecological protection, thereby enhancing the resistance and resilience of ecosystems (32). Meanwhile, public environmental awareness can guide green consumption behavior and promote the popularization of environmental values, laying a foundation for collaborative governance and social participation in ecological risk management and further improving the adaptability and learning capacity of the system (11, 33). Culture-tourism integration policies inherently function as platforms for environmental education. By embedding ecological knowledge and environmental values into tourism experiences and cultural products, they effectively establish a social transmission mechanism of “policy guidance–experience enhancement–behavior transformation.” This process not only improves public ecological awareness and emotional identification but also strengthens environmental responsibility and stimulates the endogenous motivation of social actors to participate in ecological governance (13, 33). Such policy-driven changes in social consciousness help form a collaborative and shared ecological governance pattern, thereby systematically strengthening urban ecological resilience (11). Accordingly, we propose the following hypothesis.

*Hypothesis 4*: Culture-tourism integration policies strengthen urban ecological resilience by increasing public environmental awareness.

Notably, the impacts of culture-tourism integration policies on urban ecological resilience are unlikely to follow a simple linear pattern. Instead, they may present threshold effects and contextual boundary conditions shaped by urban economic development level, resource endowments, and administrative hierarchy. For instance, the policy effects may be stronger in regions with relatively lower ecological baselines or greater development potential, while constrained in areas with strict protection requirements or saturated governance effects. Similarly, the roles of industrial structure upgrading, green technology innovation, and public environmental awareness may also vary across different urban groups, indicating nonlinear and heterogeneous transmission mechanisms.

## 3 Research design

### 3.1 Model specification

Identifying the causal effect of culture-tourism integration policies requires a rigorous econometric paradigm. Given that culture-tourism integration policies were implemented in a staggered manner across cities, this study adopts a multi-period difference-in-differences model with two-way fixed effects. By comparing the differential changes before and after policy implementation between the treatment group (policy-implementing cities) and the control group (non-policy-implementing cities), this framework effectively identifies the net policy effect. The specific model specification is shown in Equation 1:


$$ER_{i,t}=\alpha +\mathrm{\beta DI}D_{i,t}+\text{γControl}\_\mathrm{Va}r_{i,t}+\text{CityFE}+\text{YearFE}+\varepsilon_{i,t}$$ (1)

where ER denotes urban ecological resilience, DID represents the culture-tourism integration policy dummy variable, and Control_Var is a vector of control variables. City_FE and Year_FE denote city and year fixed effects, respectively, and *ε* is the random error term. The estimated coefficient *β* captures the average change in urban ecological resilience caused by the culture-tourism integration policy shock. All regressions report standard errors clustered at the city level to account for potential serial correlation within cities.

### 3.2 Variable definition

#### 3.2.1 Dependent variable

Ecological Resilience (ER). Some scholars measure ecological resilience from two perspectives: the internal development demand of the ecosystem (landscape background) and external risk response in landscape ecology (34–36). However, such methods mostly focus on descriptions of static landscape patterns and fail to fully integrate the external pressures on the ecosystem and the internal active response mechanisms into a unified framework, making it difficult to comprehensively capture the dynamic resilience of the urban complex ecosystem (37). Therefore, this paper follows the method of Chu et al. (38) and constructs an evaluation index system for urban ecological resilience from three dimensions—pressure resilience, state resilience, and response resilience—based on the Pressure-State-Response framework. The entropy method, which is widely used in economic evaluation, is adopted for objective weighting and measurement (Table 1).

**Table 1:** Urban ecological resilience evaluation index system.

First-level index	Second-level index	Unit	Attribute	Weight
Pressure	Per capita industrial wastewater discharge	Tons/person	Negative	0.0159
	Per capita industrial sulfur dioxide emission	Tons/person	Negative	0.0603
	Per capita industrial soot emission	Tons/person	Negative	0.1000
	Per capita industrial nitrogen oxides emission	Tons/person	Negative	0.2024
	Annual average PM2.5 concentration	Micrograms/cubic meter	Negative	0.0348
State	Built-up area green coverage rate	%	Positive	0.1758
	Per capita park green space area	Hectares/10,000 people	Positive	0.0154
	Per capita built-up area	Square kilometers/10,000 people	Positive	0.0159
	Per capita water resources availability	Cubic meters/person	Positive	0.0175
Response	Industrial sulfur dioxide removal volume	Tons	Positive	0.1124
	Industrial soot removal volume	Tons	Positive	0.0987
	Harmless treatment rate of domestic waste	%	Positive	0.0291
	Centralized treatment rate of wastewater treatment plants	%	Positive	0.0246
	Comprehensive utilization rate of industrial solid waste	%	Positive	0.0973

#### 3.2.2 Core independent variable

Culture-Tourism Integration Policy (DID). This study treats the implementation of culture-tourism integration policies in each city as a quasi-natural experiment. The national institutional reform in 2018 elevated the integration of culture and tourism to an important national top-level design, which provided unified policy guidance and institutional impetus for local governments. In our empirical setting, the city-level policy variable identifies the actual timing when each prefecture-level city formally issued and implemented its own specialized culture-tourism integration policy. The national top-down design and local staggered implementation are logically consistent and complementary, which helps ensure the relative exogeneity and validity of the policy shock. Following the method of Zhou et al. (39), we construct a time-varying dummy variable for culture-tourism integration policies. Policy data were primarily collected from the official websites of local governments and official media platforms. Through the “government information disclosure” module, we retrieved policy documents containing keywords such as “culture and tourism,” “integration,” “culture,” and “tourism.” We strictly retained specialized policies that explicitly included “culture-tourism integration” or highly relevant expressions in their titles. The DID variable is coded as 1 starting from the year when a city first issued the policy, and 0 otherwise. Detailed policy information and the complete city-year panel of policy implementation are available in Supplementary Data 1.

#### 3.2.3 Control variables

Following existing studies including Zhang et al. (8), Peng (40), Hu et al. (30), and Zhao et al. (10), this study further controls for city-level variables that may influence ecological resilience.

Economic development level (PGDP). Regions with higher economic development usually have stronger ecological governance capacity and greater investment in green development, but may also face intensified ecological pressure due to production and consumption activities. Economic development level is measured as the logarithm of price-deflated per capita GDP.

Population density (PD). Population density affects regional resource consumption and the scale of pollution emissions, imposing direct constraints on ecological resilience. It is measured as the ratio of year-end permanent urban population to urban administrative area.

Urbanization level (URBAN). Land use changes, infrastructure construction, and public service provision during urbanization directly affect ecosystem stability. Urbanization level is measured as the ratio of urban population to total permanent resident population.

Economic openness (OPEN). Economic openness may bring advanced technologies and management expertise to promote urban economic development, but may also introduce high-pollution and high-energy-consumption industries, exerting negative environmental impacts. The level of economic openness is measured as the ratio of total import and export volume to GDP.

Environmental regulation intensity (ERI). Following Chen and Chen (41), Deng and Yang (42), and Yin and Wu (43), this study utilizes Python to conduct word frequency statistics on environmental protection terms in prefecture-level municipal government work reports, aiming to characterize local governments’ emphasis on environmental governance and regulatory intensity.

Forest coverage rate (FOREST). Forest coverage represents regional natural ecological endowment and determines the ecosystem’s primary foundation and anti-interference capacity. It is measured as the proportion of regional forest area to total land area.

#### 3.2.4 Mechanism variables

In line with the theoretical mechanism analysis above, this study selects industrial structure upgrading (IS), green technology innovation (TECH), and public environmental awareness (PEA) as mechanism variables to identify the channels through which culture-tourism integration policies affect urban ecological resilience. Referring to Yang et al. (44), industrial structure upgrading is proxied by the share of the value added of the tertiary industry in GDP. Following Fang and Meng (45), green technology innovation is measured as the natural logarithm of the number of granted green invention patents. In line with Wu et al. (46), public environmental awareness is measured using the annual average Baidu Index for “haze (smog),” a widely used proxy for general environmental concern due to its high public salience. Definitions of the key variables are summarized in Table 2.

**Table 2:** Definition of main variables.

Type	Name	Symbol	Definition
Dependent variable	Ecological resilience	ER	Ecological resilience index measured comprehensively by the entropy method
Core independent variable	Culture-tourism integration policy	DID	Dummy variable (0–1), defined according to whether a city has issued a culture-tourism integration policy
Control variable	Economic development level	PGDP	ln(per capita GDP)
	Population density	PD	Permanent urban population at year-end/Urban area
	Urbanization level	URBAN	Urban population/total permanent urban population
	Economic openness	OPEN	Total import and export volume/GDP
	Environmental regulation intensity	ERI	Word frequency of environmental protection terms/total words in government work reports
	Forest coverage rate	FOREST	Forest area/total land area
Mechanism variables	Industrial structure upgrading	IS	Proportion of tertiary industry added value in GDP
	Green technology innovation	TECH	ln(1 + number of granted green invention patents)
	Public environmental awareness	PEA	Annual average of Baidu Index for “haze (smog)” related searches

### 3.3 Data sources and descriptive statistics

Considering that China’s culture-tourism integration policies entered a stage of systematic promotion starting in 2010 (39), this study sets the sample period to 2010–2023. Meanwhile, this study period covers exogenous shock scenarios such as major public health emergencies, which enables a valid examination of the impact of culture-tourism integration policies on ecological resilience under disturbed environments. Relevant data were mainly obtained from official statistical yearbooks issued by the National Bureau of Statistics, including the *China City Statistical Yearbook, Regional Economic Statistical Yearbook, Energy Statistical Yearbook*, as well as statistical yearbooks and statistical bulletins of prefecture-level cities. Green patent grants are obtained from the State Intellectual Property Office of China. Environmental regulation intensity is constructed by calculating the word frequency of environment-related terms in municipal government work reports using Python. The environment-related terms include: environmental protection, environmental pollution, energy consumption, emission reduction, pollution discharge, ecology, green, low-carbon, air, COD, SO₂, CO₂, PM10, and PM2.5. Baidu Index data are collected from Baidu, the largest Chinese search engine with wide coverage and high data availability. Public environmental awareness is measured based on search frequency and geographic location. In addition, to eliminate the influence of price fluctuations, this study adopts 2010 as the base year to deflate all nominal indicators involving monetary values. Descriptive statistics for all variables are presented in Table 3.

**Table 3:** Descriptive statistics.

Variables	Observations	Mean	Standard deviation	Minimum	Maximum
ER	3,976	0.3143	0.0141	0.1357	0.4654
DID	3,976	0.3632	0.4810	0	1.0000
PGDP	3,976	10.7639	0.5779	8.5762	12.4864
PD	3,976	5.7461	0.9281	1.6193	8.1756
URBAN	3,976	0.3963	0.2128	0.0750	1.000
OPEN	3,976	0.0024	0.0027	0	0.0294
ERI	3,976	0.0081	0.0029	0.0011	0.0261
FOREST	3,976	0.4050	0.0701	0	1.0000
IS	3,976	0.4338	0.1079	0.0976	0.9301
TECH	3,976	2.7398	1.8137	0	9.3936
PEA	3,976	39.8062	60.6979	0	1118.2080

## 4 Empirical results analysis

### 4.1 Baseline regression results

This study employs a two-way fixed effects difference-in-differences model, estimated using Stata 18, to evaluate the policy effects. The baseline regression results are reported in Table 4. Column (1) presents the estimates including only the core independent variable along with city and year fixed effects. Column (2) reports the results after further controlling for a set of city-level covariates. The results indicate that the estimated coefficients of DID are significantly positive at the 1% level in both specifications: 0.0066 without control variables and 0.0050 with control variables. This suggests that after accounting for city-specific heterogeneity, time trends, and other confounding factors, the implementation of culture-tourism integration policies exerts a significant positive effect on urban ecological resilience. In other words, culture-tourism integration policies can effectively improve urban ecological resilience, providing empirical support for Hypothesis 1. The estimated coefficient of the DID term is 0.0050, indicating that the culture-tourism integration policy increases urban ecological resilience by approximately 1.59% relative to its sample mean, which is economically meaningful in the context of urban ecological governance. Regarding the control variables, the coefficients of economic development level, population density, and forest coverage are significantly positive at the 1% level; the coefficient of economic openness is significantly positive at the 5% level; and the coefficient of urbanization level is significantly positive at the 10% level. The underlying economic logic is as follows: Cities with higher economic development tend to have stronger investment capacity and governance effectiveness in ecological conservation. Higher population density improves the efficiency of resource agglomeration and ecological governance. Urbanization advancement facilitates the improvement of public services and supporting ecological infrastructure. Greater economic openness contributes to the introduction of green technologies and advanced environmental management experience. Higher forest coverage indicates better ecological endowment and a more solid foundation for ecological resilience. The coefficient of environmental regulation intensity is negative but statistically insignificant, implying that the direct impact of standalone environmental regulation on urban ecological resilience is not significant. Its effect may be characterized by time lags or nonlinearity, or may need to be combined with culture-tourism integration policies to achieve effective synergy.

**Table 4:** Baseline regression results.

Variables	(1)	(2)
DID	0.0066*** (6.70)	0.0050*** (5.34)
PGDP		0.0189*** (7.37)
PD		0.0894*** (10.32)
URBAN		0.0096* (1.71)
OPEN		0.7360** (2.48)
ERI		−0.0918 (−0.61)
FOREST		0.0307*** (2.73)
Constant	0.4941*** (1101.22)	0.2407*** (4.02)
Time fixed effects	Yes	Yes
City fixed effects	Yes	Yes
*n*	3,976	3,976
Adj R-squared	0.9805	0.9820

*T*-values based on robust standard errors are reported in parentheses. ***, **, and * indicate significance at the 1, 5, and 10% levels, respectively.

### 4.2 Parallel trend assumption assessment and dynamic effect analysis

The validity and unbiasedness of the multi-period difference-in-differences model rely on the parallel trends assumption, which requires that urban ecological resilience in the treatment and control groups follows similar trajectories before the implementation of culture-tourism integration policies. Given the heterogeneity in the timing of policy adoption across cities, this study does not define dummy variables based on a single time point; instead, we construct variables according to the relative time of policy implementation in each city. Therefore, this study adopts the event study approach to conduct the parallel trend test and capture dynamic effects. The specific model specification is shown in Equation 2:


$$\begin{array}{c} ER_{i,t}=\alpha +\beta_{1}\text{Before}3_{i,t}+\beta_{2}\text{Before}2_{i,t}+\beta_{3}\text{Before}1_{i,t}\\ +\beta_{4}\text{Curren}t_{i,t}+\beta_{5}\text{After}1_{i,t}+\beta_{6}\text{After}2_{i,t}\\ +\beta_{7}\text{After}3_{i,t}+\text{γControl}\_\mathrm{Va}r_{i,t}+\text{CityFE}\\ +\text{YearFE}+\varepsilon_{i,t} \end{array}$$ (2)

**Figure 1:**
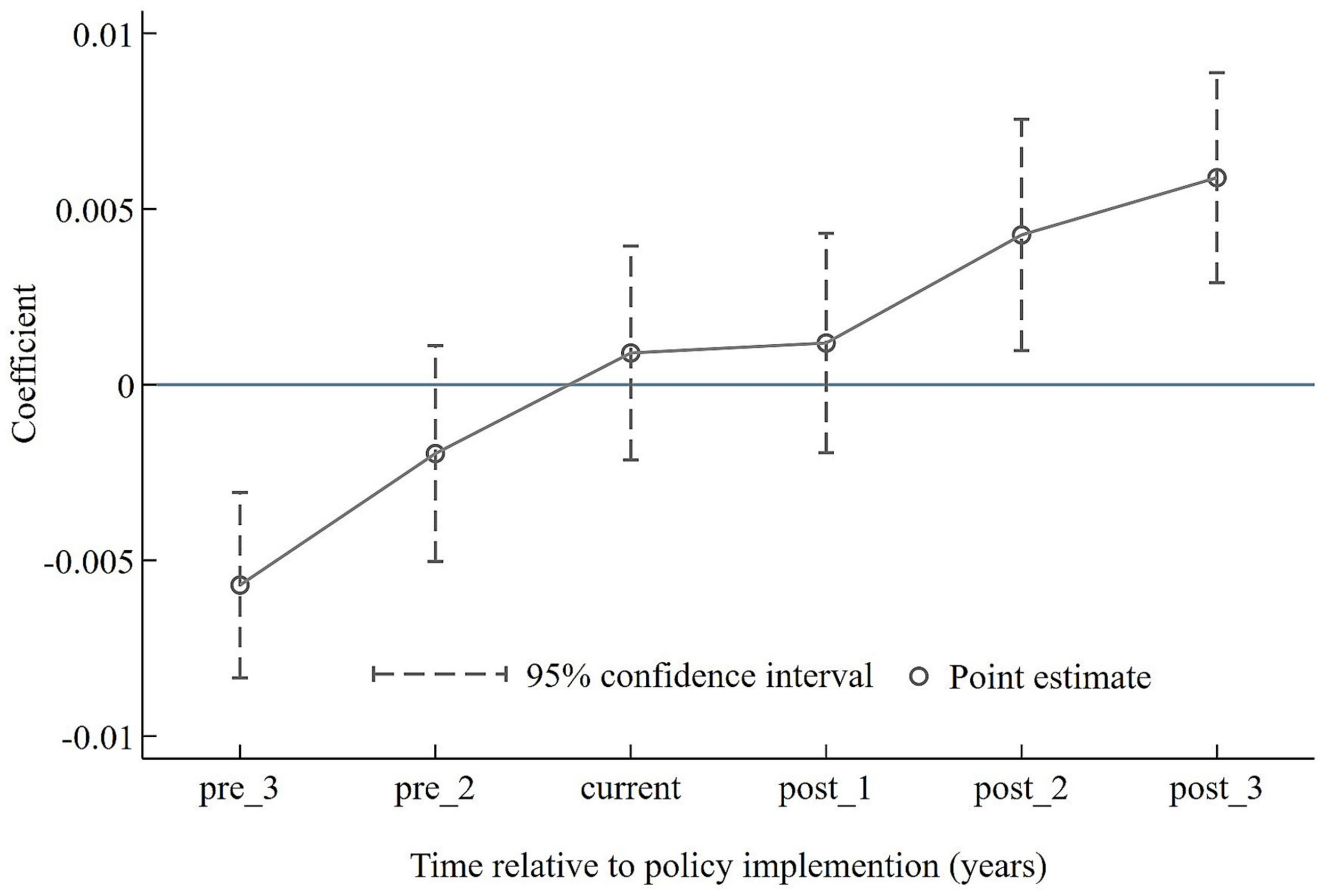
Parallel trend test of the culture-tourism integration policy. The dashed lines above and below the hollow dots represent the 95% confidence intervals.

Specifically, time dummy variables are constructed according to the relative timing of policy implementation in each city, covering 3 years before, the year of, and 3 years after the culture-tourism integration policy. Accordingly, for cities that have not implemented such policies, all time dummy variables are coded as 0 throughout. The sample period of this study spans 2010–2023. Due to the uneven distribution of policy implementation years, the sample size for periods earlier than or equal to −4 before policy adoption is insufficient in some cities. To avoid multicollinearity, the period 4 years before policy implementation (pre_4) is set as the omitted reference period. Finally, dummy variables are constructed for 3 years before, the year of, and 3 years after policy implementation for the event study analysis. Among them, the dummy variable for cities that have not implemented the policy is coded as 0. The estimated coefficients and their 95% confidence intervals are plotted in Figure 1. To facilitate rigorous statistical inference, Table 5 reports the specific coefficients, standard errors, and significance levels corresponding to the event study estimation. The definitions and treatments of other explanatory variables are consistent with those in the baseline model to ensure the robustness of the results. The event-study results show that most of the pre-policy coefficients are statistically insignificant and fluctuate around zero. Although the coefficient of pre_3 is statistically significant, no systematic upward or downward trend is detected in the pre-policy periods. Overall, the evidence generally supports the parallel trends assumption. To further justify the exogeneity of the policy shock, these results confirm that policy adoption is not driven by prior ecological conditions. City fixed effects also absorb time-invariant city characteristics such as tourism resources, fiscal capacity, governance capacity, and initial ecological status. These results support the relative exogeneity of our identification strategy. After policy implementation, the dynamic effects gradually emerge: the point estimate for post_1 is slightly above zero but the confidence interval includes zero, indicating an insignificant effect; the point estimate for post_2 becomes significantly positive with the confidence interval excluding zero, suggesting that the positive impact begins to materialize; the point estimate for post_3 rises further, indicating that the policy effect continues to strengthen. These findings reveal that the promoting effect of culture-tourism integration policies on ecological resilience is lagged and gradually intensifies over time, providing dynamic empirical support for the baseline regression results.

**Table 5:** Event study estimates for the parallel trend test.

Variables	Coefficient	Standard error	*T*-value
pre_3	−0.0024	0.0012	−2.00**
pre_2	0.0008	0.0011	0.73
pre_1	−0.0002	0.0010	−0.20
current	0.0012	0.0011	1.09
post_1	0.0016	0.0011	1.45
post_2	0.0034	0.0014	2.43**
post_3	0.0052	0.0015	3.47***
Controls	Yes		
City/year FE	Yes		
*N*	3,976		

The omitted reference period is pre_4. Standard errors are clustered at the city level. ***, **, and * indicate significance at the 1, 5, and 10% levels, respectively.

### 4.3 Robustness tests

#### 4.3.1 Time-varying PSM-DID model

**Figure 2:**
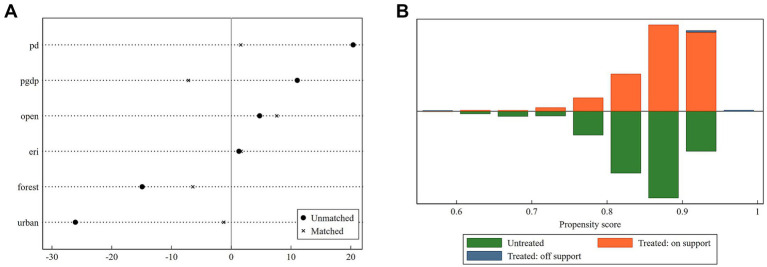
Balancing test results: **(A)** balancing test for cross-sectional PSM; **(B)** distribution of samples satisfying the common support assumption.

Although the time-varying difference-in-differences model can effectively identify the average treatment effect of policies, culture-tourism integration policy is not a strictly randomized natural experiment and thus may still suffer from sample selection bias. To address this concern, this study further constructs a time-varying propensity score matching difference-in-differences (PSM-DID) model for robustness testing. Given the limitations of conventional PSM in panel data matching, this study follows existing literature and adopts two matching strategies (47, 48): cross-sectional PSM and year-by-year matching. Specifically, the matching covariates include economic development level, population density, economic openness, environmental regulation intensity, and forest coverage rate. We first adopt the logit regression framework to estimate propensity scores. In this study, we select nearest-neighbor matching with a caliper of 0.01 and apply matching without replacement to guarantee matching accuracy and avoid repeated sample matching bias. Cross-sectional PSM treats panel data as a single cross-section for matching, while year-by-year matching conducts matching annually. Both approaches are used to select the optimal control group that satisfies the common support condition for cities implementing culture-tourism integration policies. After conducting the balancing test for the matched samples generated by the two methods, the time-varying difference-in-differences model is applied again to evaluate the impact of culture-tourism integration policy on urban ecological resilience. In addition, we further compare the sample scale changes before and after matching, and the results verify that the sample sizes of the treatment group and control group are reasonably adjusted and reach effective equilibrium after matching. Figure 2A presents the balancing test results after matching the panel data as cross-sectional data. The standardized mean differences of all covariates are less than 10% after matching, which are substantially lower than those before matching. More strictly, most standardized biases of matched covariates are reduced to below 5%, fully meeting the high-standard balance requirement. This indicates that no significant differences exist between the treatment and control groups in terms of matching covariates after matching, thereby satisfying the requirements of the balancing test. Figure 2B shows the distribution of propensity scores under the common support condition. The propensity scores of the treatment and control groups overlap significantly within the range of 0.7 to 0.9, suggesting that cities implementing culture-tourism integration policy can be well matched with counterparts in the control group, which validates the common support assumption. The distribution of propensity scores becomes much closer between the two groups after matching, further confirming that the matched samples are well balanced. Columns (1) and (2) in Table 6 report the regression results of the time-varying PSM-DID under the two matching methods. The results show that the coefficients of DID remain significantly positive, with no substantial divergence from the baseline regression. This confirms, to a certain extent, that the enhancing effect of culture-tourism integration policy on urban ecological resilience is robust.

**Table 6:** Robustness tests.

Variables	(1)	(2)	(3)	(4)	(5)	(6)
	Cross-sectional PSM	Year-by-year PSM	Lagged explanatory variable	Excluding confounding policy effects 1	Excluding confounding policy effects 2	Changing the sample period
DID	0.0051*** (3.70)	0.0053*** (3.73)	0.0036*** (3.89)	0.0050*** (5.33)	0.0043*** (2.54)	0.0066*** (6.19)
DID1				0.0014 (1.02)		
DID2					0.0233 (0.46)	
Control variables	Yes	Yes	Yes	Yes	Yes	Yes
Year fixed effects	Yes	Yes	Yes	Yes	Yes	Yes
City fixed effects	Yes	Yes	Yes	Yes	Yes	Yes
Adj R-squared	0.2075	0.2073	0.9814	0.9805	0.9365	0.9804

*T*-values based on robust standard errors are reported in parentheses. ***, **, and * indicate significance at the 1, 5, and 10% levels, respectively.

#### 4.3.2 Lagged independent variable

To address potential endogeneity concerns arising from the possible impact of culture-tourism integration policies on contemporaneous control variables and reverse causality between control variables and the policy variable, this study conducts a robustness test by lagging the independent variable by one period. Specifically, the regression is re-estimated with the culture-tourism integration policy variable lagged by 1 year. As shown in Column (3) of Table 6, the sign and statistical significance of the policy variable remain broadly consistent with the baseline regression. This finding strongly supports the robustness of the main results and indicates that the empirical findings are not significantly affected by potential endogeneity.

#### 4.3.3 Excluding confounding policy effects

During the sample period, the National Civilized City Selection policy (DID1), implemented since 2005, may indirectly influence ecological resilience by shaping urban environmental governance and public services. Meanwhile, the National Culture and Tourism Consumption Pilot Cities program (DID2), launched during 2021–2022, directly targets the culture and tourism sector and may generate interactive effects with culture-tourism integration policies. To rule out confounding effects from these policies, this study sequentially adds dummy variables for the implementation years of the two aforementioned policies to the baseline model. The results, reported in Columns (4) and (5) of Table 6, show that the coefficient of the culture-tourism integration policy dummy remains significantly positive after controlling for these policies, confirming the robustness of the enhancing effect on urban ecological resilience. Culture-tourism integration policies upgrade the urban ecological environment and strengthen ecological protection awareness by integrating cultural and tourism resources, thereby improving urban ecological resilience.

#### 4.3.4 Changing the sample period

The operation of the culture and tourism industry heavily relies on cross-regional mobility and offline gatherings. However, the recent major public health emergency disrupted this normal pattern, and strict mobility restrictions imposed severe external shocks on the tourism market. Although the baseline estimation absorbs macro time-varying shocks via year fixed effects, this study further excludes observations from 2020 and 2021 to account for extreme fluctuations in those years and re-estimates the model to verify the robustness of the core findings. As shown in Column (6) of Table 6, the coefficient of the core independent variable remains highly significantly positive. This suggests that the effect of culture-tourism integration policies in improving urban ecological resilience remains solid after excluding the period of extreme external shocks, further corroborating the reliability of the baseline results.

#### 4.3.5 Placebo test

**Figure 3:**
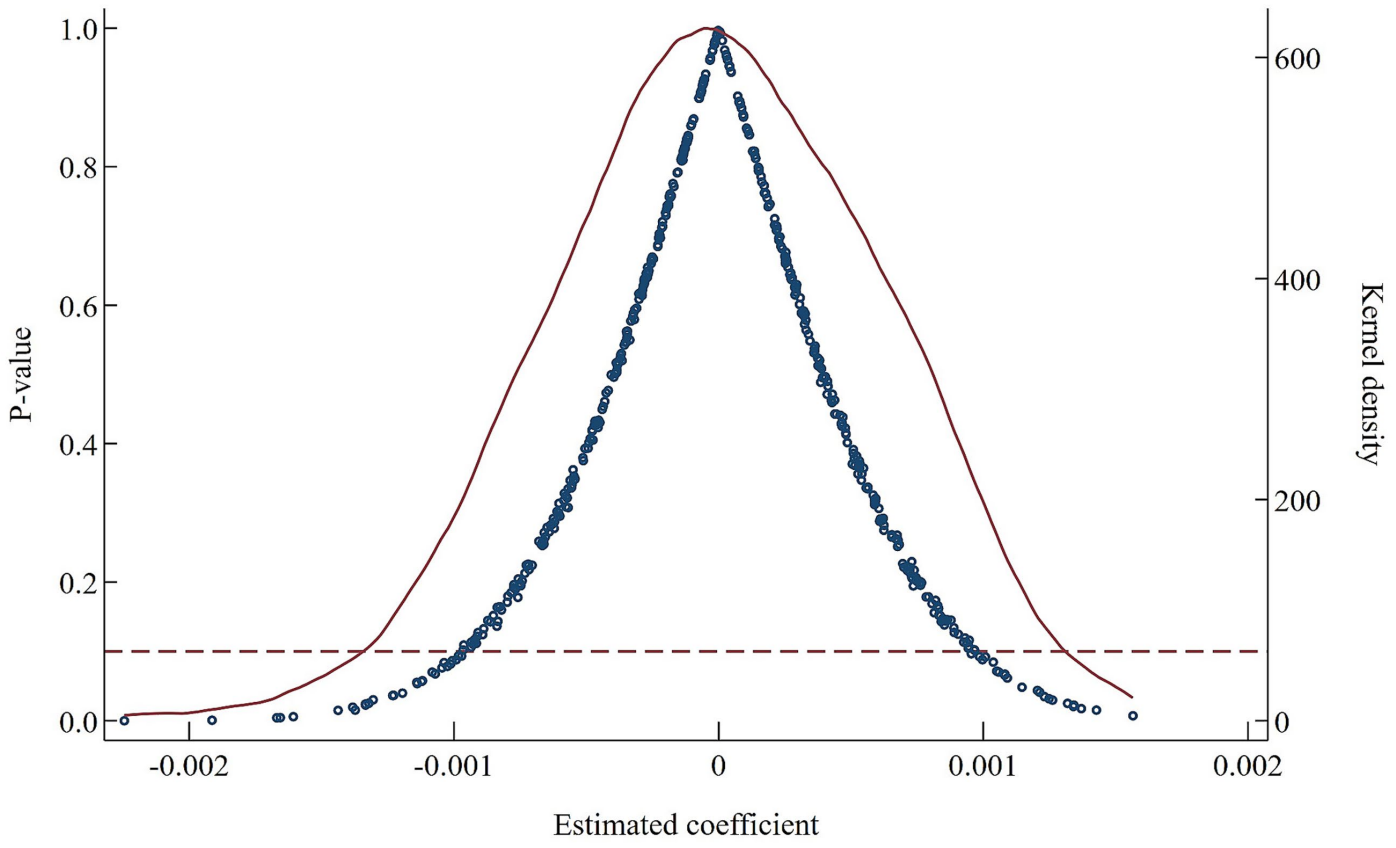
Distribution of coefficient estimates after randomization inference. The *X*-axis shows the estimated coefficients of DID_random generated from 500 random permutations. The open circles represent the *p*-values of the estimated coefficients, and the solid line denotes the kernel density distribution of the estimated coefficients.

To eliminate interference from unobservable city characteristics or random factors in assessing the effect of culture-tourism integration policies, this study conducts a placebo test to rule out spurious estimation results. Following Bai et al. (49), we use Stata 18 to generate 500 random pseudo-policy shocks for culture-tourism integration across the 284 sample cities, creating 500 sets of the dummy variable DID_random. Specifically, sample cities are randomly assigned to a pseudo-treatment group with randomly set policy shock timings. We then re-estimate Model (1) and examine the distribution of coefficients and *p*-values, with results shown in Figure 3. The estimated coefficients of DID_random are mainly concentrated around zero, and the kernel density curve shows a symmetric distribution centered at zero. Meanwhile, most p-values corresponding to the DID_random coefficients are greater than 0.10 and insignificant at the 10% level, indicating that the randomly generated pseudo-policy effects are not statistically significant. In contrast, the estimated coefficient of the actual culture-tourism integration policy (0.0050, as reported above) is distinctly different from the placebo test results. This indicates that the empirical findings of this study are not significantly affected by potential random factors, and the baseline conclusions are robust.

### 4.4 Endogeneity discussion

#### 4.4.1 Panel instrumental variable approach

Although this study constructs a model based on exogenous policy shocks and controls for multi-dimensional city-level characteristics as comprehensively as possible, it remains difficult to completely avoid the interference of endogeneity issues. In particular, the implementation of culture-tourism integration policies and urban ecological resilience may be subject to reverse causality, leading to estimation bias. To address endogeneity concerns caused by potential omitted variables and reverse causality, this study further adopts the instrumental variable (IV) approach.

Because topographic slope difference is a time-invariant static geographic feature that cannot capture time-varying variation in panel data, we construct an IV by interacting the topographic slope difference with the national total tourism revenue growth rate of the previous year, following the identification strategy of Nunn and Qian (50). The rationality of this IV is justified as follows:

First, regarding the relevance assumption: the topographic slope difference directly constrains the difficulty and spatial layout of local tourism development. The impact of the overall expansion of the national tourism market is conditioned by topographic factors; regions with gentle terrain can better absorb resources during macro tourism booms. The interaction term effectively captures the urban culture-tourism development potential under specific geographic constraints, and is thus highly correlated with the adoption and implementation intensity of culture-tourism integration policies, satisfying the relevance requirement of instrumental variables.

Second, regarding the exclusion restriction: the topographic slope difference is an objective and stable natural geographic endowment, and the national tourism revenue growth rate is a macro exogenous shock to individual cities. The interaction term is unlikely to be driven by the socioeconomic characteristics of a single city. While urban ecological resilience depends heavily on localized factors such as economic structure and environmental governance, we acknowledge that the instrumental variable constructed from topographic slope may still affect urban ecological resilience through natural ecological conditions, which means the exclusion restriction of this IV relies on a relatively strong assumption. Nevertheless, the interaction of objective topographic differences and national macro trends is unlikely to constitute a systematic channel that directly affects a specific city’s ecological resilience except through the culture-tourism integration policies, thus largely satisfying the exclusion restriction. Given this strong assumption, we treat the IV estimation results as supplementary robustness evidence rather than definitive causal evidence. Although the interaction with macro-level exogenous trends substantially weakens the endogeneity concern, we transparently acknowledge that the exclusion restriction cannot be definitively proven. Topographic features may theoretically influence ecological resilience through natural geographic channels independent of the policy. Therefore, we refrain from over-relying on the IV coefficient magnitude and consider it as supplementary evidence confirming the direction and significance of the policy effect.

Table 7 summarizes the endogeneity test results utilizing the IV strategy. Diagnostic checks confirm that the chosen IV is both relevant and strong; for instance, the first-stage regression yields an *F*-statistic of 13.32 (well above the conventional rule-of-thumb of 10), demonstrating a robust link with the endogenous regressor. Potential identification failures are ruled out, as evidenced by the Kleibergen-Paap rk LM test (*p* = 0.0311) and a Wald rk *F*-statistic (25.42) that surpasses the Stock-Yogo critical value of 16.38. Crucially, when transitioning to the second-stage estimation to correct for endogeneity, the policy variable retains its positive and statistically significant influence on ecological resilience (coefficient = 0.1424 at the 5% level). The much larger coefficient in the IV estimation reflects the local average treatment effect. The instrumental variable identifies the policy effect for cities whose adoption of culture-tourism integration policies is affected by terrain conditions. These cities are more suitable for tourism development, so the policy effect is stronger. By contrast, the baseline difference-in-differences estimate shows the average treatment effect for the full sample. The difference between the two estimates is expected and common in the IV approach. Nonetheless, the consistency of the IV results with the baseline estimates firmly reinforces the reliability of our core conclusions.

**Table 7:** Endogeneity test based on the instrumental variable method.

Variables	First-stage results	Second-stage results
	(1)	(2)
	DID	ER
Instrumental variable	0.0007** (2.18)	
DID		0.1424** (2.13)
Kleibergen-Paap rk LM statistic	4.65 [0.0311]	
Kleibergen-Paap Wald rk *F* statistic	25.42 {16.38}	
Control variables	Yes	Yes
City fixed effects	Yes	Yes
Year fixed effects	Yes	Yes
*n*	3,692	3,692
*F* statistic	13.32	

Values in [] are p-values; values in {} are the Stock-Yogo critical values at the 10% level; values in () are t-statistics based on robust standard errors.

To further verify the exclusion restriction, we conducted a balancing test and a pre-policy placebo test. The balancing test shows the IV is uncorrelated with predetermined covariates (Supplementary Table A1). The pre-policy placebo test shows the IV does not affect ER before the policy implementation (Supplementary Table A2). These statistical checks support the validity of the exclusion restriction.

#### 4.4.2 Double machine learning

Although the previous analysis has controlled for omitted variables to the greatest extent, traditional linear models still suffer from two key limitations. First, model specification bias may exist because the true data-generating process may involve complex nonlinear relationships and interaction effects that cannot be fully captured by conventional parametric models. Second, the inclusion of numerous control variables may lead to overfitting, which undermines the consistency of causal estimates. To address these concerns, this study employs the double machine learning framework proposed by Chernozhukov et al. (51) for re-estimation. This approach offers two distinct advantages. On the one hand, it adopts nonparametric machine learning algorithms such as Random Forest (RF) and Gradient Boosting (GB) to flexibly accommodate potential high-order terms and nonlinear correlations between covariates and the core explanatory variable, thereby avoiding artificial model misspecification. On the other hand, through sample splitting and cross-fitting, double machine learning separates variable selection from causal inference, effectively mitigating the regularization bias inherent in machine learning algorithms and yielding unbiased causal effect estimates.

Specifically, we set the number of folds for sample splitting to *K* = 5. In the auxiliary regressions, three algorithms—RF, LASSO, and GB—are used to fit the residuals of the core explanatory variable and the dependent variable. Causal effects are then estimated based on the residualized components. The results are reported in Table 8. Columns (1)–(3) show that after accounting for complex nonlinear relationships and alleviating overfitting, the coefficient of culture-tourism integration policy on ecological resilience remains significantly positive at the 1% level, confirming the strong robustness of the baseline findings. To further examine the sensitivity of the results to algorithm selection and sample splitting, we conduct two additional robustness checks: (1) changing the cross-fitting folds to *K* = 2 and *K* = 10; (2) replacing the machine learning estimators with Support Vector Machine (SVM) and Neural Network (NN). The re-estimated results [Columns (4)–(7)] indicate that the coefficients of the core policy variable remain significantly positive, with no material changes in the direction or statistical significance. The slight numerical differences in coefficients among LASSO, RF, and GB are inherently determined by the algorithmic characteristics of different machine learning models. Specifically, the LASSO algorithm emphasizes variable sparsity and feature selection, which tends to screen out weakly correlated covariates and retain core influencing factors, thus yielding relatively conservative estimation results. In contrast, RF and GB algorithms possess outstanding advantages in capturing high-order nonlinear relationships and complex interaction effects among variables, which can better fit the implicit nonlinear patterns in real economic data and produce slightly different coefficient magnitudes. Despite the numerical differences caused by algorithm heterogeneity, all estimation results across the three approaches consistently show significantly positive coefficients of culture-tourism integration policies. The consistent positive sign and statistical significance fully verify that the promotional effect of culture-tourism integration on urban ecological resilience is not dependent on the selection of specific machine learning algorithms. This further strongly confirms the robustness and credibility of our core research conclusions.

**Table 8:** Double machine learning analysis.

Variables	(1)	(2)	(3)	(4)	(5)	(6)	(7)
	RF (*K* = 5)	LASSO	GB	RF (*K* = 2)	RF (*K* = 10)	SVM	NN
DID	0.0072*** (0.0015)	0.0046*** (0.0009)	0.0305*** (0.0034)	0.0094*** (0.0018)	0.0053*** (0.0005)	0.0453*** (0.0037)	0.0049*** (0.0016)
Linear terms of controls	Yes	Yes	Yes	Yes	Yes	Yes	Yes
Quadratic terms of controls	Yes	Yes	Yes	Yes	Yes	Yes	Yes
Year fixed effects	Yes	Yes	Yes	Yes	Yes	Yes	Yes
City fixed effects	Yes	Yes	Yes	Yes	Yes	Yes	Yes
n	3,976	3,976	3,976	3,976	3,976	3,976	3,976

Robust standard errors are reported in parentheses. *, **, and *** denote significance at the 10, 5, and 1% levels, respectively.

### 4.5 Further discussion on staggered difference-in-differences estimation

Recent studies have shown that the traditional two-way fixed effects estimator may be biased in staggered difference-in-differences designs when treatment effects are heterogeneous across cohorts. To assess this concern, we examine the dynamic effects from the event study, which display a stable and gradually increasing pattern without substantial heterogeneity across policy implementation cohorts. In addition, a series of robustness checks, including placebo tests and PSM-DID, all support the consistency of the baseline results. Therefore, while we cannot fully rule out the potential bias of the two-way fixed effects estimator under staggered treatment settings, the stable dynamic pattern observed in the event study and consistent results across a battery of robustness checks collectively suggest that our core findings are still plausible and informative.

Recent econometric advancements highlight that the traditional two-way fixed effects estimator may yield biased estimates under heterogeneous treatment effects in staggered DID designs, potentially generating “forbidden comparisons” and negative weights. We acknowledge this potential limitation. However, we argue that our baseline estimates remain highly plausible for two reasons. First, the event-study dynamic effects (Figure 1) display a stable, monotonically increasing pattern without substantial heterogeneity across policy implementation cohorts, which mechanically mitigates the concern of severe negative weighting. Second, our baseline conclusions are robustly supported by the PSM-DID approach and the Double Machine Learning framework. The Double Machine Learning approach, in particular, flexibly accommodates non-linear relationships and complex interactions, providing a non-parametric robustness check that is less susceptible to the specific biases of the two-way fixed effects estimator. Future research could employ newly developed heterogeneous treatment effect estimators to further validate these findings.

### 4.6 Mechanism analysis and heterogeneity analysis

#### 4.6.1 Mechanism tests

The baseline regression and robustness tests consistently confirm that culture-tourism integration policies significantly improve urban ecological resilience. To further clarify the underlying transmission channels of the policy effects, this study follows the mediation effect identification strategy proposed by Jiang (52) and constructs a multi-period difference-in-differences model to examine the impacts of culture-tourism integration policies on industrial structure upgrading, green technology innovation, and public environmental awareness, respectively, to verify the proposed mediating mechanisms. The results are reported in Table 9.

**Table 9:** Mechanism test results.

Variables	IS	TECH	PEA
	(1)	(2)	(3)
DID	0.0015*** (3.58)	0.0081*** (5.68)	1.4019** (2.16)
Control variables	Yes	Yes	Yes
Year fixed effects	Yes	Yes	Yes
City fixed effects	Yes	Yes	Yes
*n*	3,976	3,976	3,976
R-squared	0.8284	0.9085	0.8237

*T*-statistics based on robust standard errors are reported in parentheses. ***, **, and * indicate significance at the 1, 5, and 10% levels, respectively.

Industrial structure upgrading reflects the policy-induced structural transformation of the economic system. Column (1) shows that the coefficient of the culture-tourism integration policy is 0.0015 and significant at the 1% level, indicating that the policy effectively promotes the upgrading of the industrial structure. Specifically, the policy strengthens regional ecological constraints and raises environmental entry standards, which gradually force traditional high-energy-consuming and high-pollution industries to exit from urban core areas. Meanwhile, culture-tourism integration drives the agglomeration of green services, cultural creativity, and low-carbon supporting industries. In this way, the policy promotes both the rationalization of factor allocation and the advancement of industrial structure. This supports the factor reallocation mechanism: the policy breaks path dependence and directs capital, technology, and other factors toward modern culture-tourism service industries. Such structural changes reduce ecological pressure in production and drive the green upgrading of the industrial chain through industrial linkages, thereby enhancing the resistance and resilience of the urban ecosystem. The empirical results support Hypothesis 2.

Green technology innovation captures the policy’s inductive effect on the technological paradigm of production modes. Column (2) shows that culture-tourism integration policies significantly increase green innovation (coefficient = 0.0081, *p* < 0.01). The logic is that the policy establishes an incentive-compatible institutional environment, reducing R&D costs and risks for enterprises by setting green standards, providing subsidies, and creating consumption scenarios. The combination of demand-side pull and supply-side push stimulates enterprises’ endogenous motivation to adopt clean technologies and digital governance tools, improving the ecosystem’s ability to accurately identify and adapt to risks. The empirical results support Hypothesis 3.

Public environmental awareness reveals the transmission mechanism at the social cognitive level. Column (3) shows that the coefficient of the culture-tourism integration policy is 1.4019, significantly positive at the 5% level. Combined with environmental cognition theory, cultural-tourism scenarios provide vivid and immersive ecological experiences and implicit environmental education, which effectively reshape public environmental cognition, correct cognitive biases, and enhance residents’ ecological awareness and environmental responsibility at the cognitive stage. Such upgraded environmental cognition further stimulates individual pro-environmental preferences and gradually transforms into stable, long-term green lifestyles and active environmental supervision behaviors, realizing the transition from environmental cognition to individual environmental behavior. Furthermore, widespread public environmental participation forms a bottom-up social governance force that restrains unreasonable industrial and residential pollution behaviors, thereby achieving the progression from individual behavioral transformation to collaborative environmental governance. This confirms the unique “platform for environmental education” attribute of culture-tourism integration, which implicitly embeds ecological concepts into tourism experiences and cultural products, forming a social transmission chain of “policy guidance—experience enhancement—behavioral transformation.” The environmental awareness awakened among the public during culture-tourism consumption translates into concrete actions, such as monitoring corporate pollution and participating in environmental governance, which consolidates the social cognitive foundation of ecological resilience. Consequently, by constraining unreasonable human pollution behaviors, this enhanced awareness may have potential implications for anthropogenic environmental exposure intensity in residential and recreational spaces, may generate public health co-benefits, and may ultimately contribute to the coordinated improvement of ecological resilience and public health benefits. The empirical results support Hypothesis 4.

Based on the classical mechanism identification framework of Jiang, this paper focuses on testing the impact of culture-tourism integration policy on intermediate variables. The above results provide suggestive and empirical evidence for the potential influence channels through which the policy affects urban ecological resilience. This study adopts the stepwise regression method to test the mediating effects, which can effectively identify the existence and direction of mediating channels. However, this method has certain limitations: it only judges the significance of coefficients step by step, without reporting the specific magnitude of the mediating effect, its proportion in the total effect, or the Bootstrap 95% confidence interval. In line with the logic of Jiang (52), our results confirm that the above results provide supportive evidence for the potential influence channels through which the policy affects urban ecological resilience. Future research may adopt the Bootstrap method to obtain more precise statistical results of the mediating effect size and confidence intervals.

To rigorously quantify the mediating effects and meet current academic criteria, we further employ a clustered Bootstrap approach (resampling at the city level with 1,000 replications) to estimate the indirect effects, direct effects, total effects, 95% confidence intervals, and the proportion mediated. The results are reported in Supplementary Table A3. The indirect effects through IS, TECH, and PEA are 0.0012, 0.0016, and 0.0008, respectively. Crucially, their 95% confidence intervals—[0.0005, 0.0020], [0.0008, 0.0025], and [0.0002, 0.0015]—all exclude zero, confirming the statistical significance of the mediating channels. The proportions mediated are 24.0, 32.0, and 16.0%, indicating that green technology innovation plays the most dominant role in transmitting the policy’s ecological benefits. These Bootstrap results provide robust statistical evidence for the proposed mechanisms.

#### 4.6.2 Heterogeneity analysis

Given the significant disparities in geographical location, resource endowments, and administrative hierarchy across cities, the impact of culture-tourism integration policies on urban ecological resilience may exhibit an unbalanced pattern. To investigate this heterogeneity, this study conducts subsample regressions based on three dimensions: geographical location, resource endowments, and urban hierarchy. The results are presented in Table 10.

**Table 10:** Heterogeneity test results.

Variables	ER						
	(1)	(2)	(3)	(4)	(5)	(6)	(7)
	East	Central	West	National historical and cultural cities	Non-designated national historical and cultural cities	Municipalities directly under the central government and provincial capitals	Ordinary prefecture-level cities
DID	0.0027* (1.91)	0.0037** (2.18)	0.0039** (2.38)	0.0019 (1.41)	0.0072*** (5.56)	0.0113*** (3.76)	0.0044*** (4.48)
Control variables	Yes	Yes	Yes	Yes	Yes	Yes	Yes
Constant	−0.4682*** (−5.89)	−0.0405 (−0.37)	−0.1240 (−1.26)	−0.3911*** (−4.45)	−0.2329*** (−3.00)	−0.3144* (−2.03)	−0.3075*** (−4.52)
Time fixed effects	Yes	Yes	Yes	Yes	Yes	Yes	Yes
City fixed effects	Yes	Yes	Yes	Yes	Yes	Yes	Yes
*n*	1,400	1,400	1,176	1,540	2,436	420	3,556
*R*-squared	0.9843	0.9626	0.9898	0.9831	0.9794	0.9741	0.9809

*T*-statistics based on robust standard errors are reported in parentheses. ***, **, and * indicate significance at the 1, 5, and 10% levels, respectively.

##### 4.6.2.1 Heterogeneity based on geographical location

To examine the variation in policy effects across regions, the full sample is divided into Eastern, Central, and Western regions for grouped regressions [Columns (1)–(3) in Table 10]. The results show that culture-tourism integration policies exert significantly positive effects on ecological resilience across all three regions. However, the coefficient magnitudes suggest a descriptive pattern in which the estimated effect appears stronger in the western and central regions than in the eastern region. These differences are purely descriptive; no formal statistical tests for cross-group differences have been conducted. This regional difference is jointly determined by ecological endowment conditions, national policy preferences, and diminishing marginal returns. The economically developed Eastern region already possesses a more sophisticated ecological governance system and a higher baseline level of ecological resilience, leaving limited room for further improvement. In contrast, the Western and Central regions feature inherently fragile ecological backgrounds, low initial ecological resilience levels, and abundant undeveloped cultural and tourism resources, and they receive stronger national policy inclination and industrial support for cultural-tourism development. Through the mechanism of ecological value conversion, culture-tourism integration policies transform these regions’ clear waters and green mountains into invaluable assets. This not only generates economic benefits but also drives investment in ecological restoration and environmental infrastructure, leading to a more pronounced policy dividend and a significant enhancement of ecological resilience.

##### 4.6.2.2 Heterogeneity based on resource endowments

To analyze the impact of resource endowments, the sample is divided into national historical and cultural cities and non-designated national historical and cultural cities [Columns (4)–(5) in Table 10]. Descriptive results show that the policy coefficient is statistically insignificant for the former group, whereas it is 0.0072 and significant at the 1% level for the latter. This difference is a descriptive observation rather than a strict statistically significant difference between groups. This pattern may be understood from the dual lens of “improvement space” and “binding constraints.” On the one hand, national historical and cultural cities are strictly restricted by cultural heritage protection redlines and rigid environmental capacity constraints. The mandatory requirements of heritage preservation may greatly limit the development scale, industrial layout and expansion space of cultural-tourism integration, potentially imposing rigid constraints on ecological optimization. The conflict between urban cultural heritage protection and tourism development is acute, possibly making it difficult for culture-tourism integration policies to translate into incremental ecological resilience in the short term. On the other hand, although non-designated national historical and cultural cities lack abundant cultural resources, they are free from heritage institutional constraints, with weaker original ecological foundations and insufficient environmental infrastructure, suggesting huge marginal improvement potential for ecological governance. Driven by culture-tourism integration policies, these cities may tend to attract talent by constructing new ecological parks and improving green infrastructure. This “gap-filling effect” may leads to a substantial increase in ecological resilience, demonstrating obvious latecomer advantages in ecological green transformation.

##### 4.6.2.3 Heterogeneity based on urban hierarchy

To examine the moderating role of administrative hierarchy, the sample is divided into municipalities directly under the central government and provincial capital cities versus ordinary prefecture-level cities [Columns (6)–(7) in Table 10]. The regression results indicate that culture-tourism integration policies significantly improve ecological resilience in both types of cities. Descriptively speaking, the coefficient for municipalities directly under the central government and provincial capitals (0.0113) is larger than that for ordinary prefecture-level cities (0.0044). This is only a descriptive pattern and should not be interpreted as a strict statistically significant difference between groups. Combined with administrative hierarchy advantage and fiscal decentralization theory, this discrepancy stems primarily from the resource agglomeration advantage and policy implementation efficiency of high-tier cities. Municipalities directly under the central government and provincial capitals typically possess stronger fiscal capacity, more comprehensive infrastructure, and a higher-quality talent pool. These advantages enable them to respond more efficiently to culture-tourism integration policies, rapidly enhancing ecological governance through optimal resource allocation and technological diffusion. In contrast, ordinary prefecture-level cities are constrained by fiscal limitations and administrative resource shortages, leading to a relatively sluggish policy transmission mechanism and thereby dampening the policy effects to a certain extent.

Although the coefficient magnitude varies across subgroups, we regard it only as suggestive of heterogeneity for descriptive purposes. Strict statistical inference of inter-group differences requires formal coefficient difference tests, which are not further conducted in this study. Thus, we avoid overinterpreting the hierarchical gap across groups.

### 4.7 Nonlinear and moderating effect analysis

Although the subgroup regressions in Section 4.6.2 reveal heterogeneous policy effects across different city types, these discrete comparisons cannot rigorously identify continuous nonlinear relationships. To address this limitation and formally test whether the policy effect varies non-linearly with urban economic conditions, we construct a moderating effect model by introducing an interaction term between the DID variable and PGDP. The specific model specification is shown in Equation 3:


$$\begin{array}{c} ER_{i,t}=\alpha +\beta_{1}\mathrm{DI}D_{i,t}+\beta_{2}\mathrm{PGD}P_{i,t}+\beta_{3}(\mathrm{DI}D_{i,t}\times \mathrm{PGD}P_{i,t})\\ +\text{γControl}\_\mathrm{Va}r_{i,t}+\text{CityFE}+\text{YearFE}+\varepsilon_{i,t} \end{array}$$ (3)

where the core coefficient of interest is β_3_. If β_3_ is statistically significant, it indicates that the marginal effect of the culture-tourism integration policy on ecological resilience depends continuously on the level of economic development. The results are reported in Table 11. The coefficient of the interaction term (DID×PGDP) is −0.0028 and significant at the 1% level, implying that the marginal effect of the culture-tourism integration policy on ecological resilience diminishes as the level of economic development increases. Specifically, at the sample mean of PGDP (10.76), the marginal effect of the policy is 0.0039, which is highly consistent with the baseline estimate. However, for cities with one standard deviation lower PGDP, the marginal effect increases to 0.0064, while for cities with one standard deviation higher PGDP, the marginal effect decreases to 0.0014. This formally validates the descriptive finding in Section 4.6.2.1 that the policy effect is weaker in the highly developed Eastern region compared to the Central and Western regions, reflecting a nonlinear pattern of diminishing marginal returns in ecological governance.

**Table 11:** Moderating effect test based on economic development level.

Variables	ER
DID	0.0350*** (4.37)
PGDP	0.0150*** (5.00)
DID × PGDP	−0.0028*** (−2.85)
PD	0.0850*** (9.80)
URBAN	0.0085* (1.72)
OPEN	0.7200** (2.45)
ERI	−0.0850 (−0.55)
FOREST	0.0280*** (2.65)
Constant	0.1500*** (3.10)
Time fixed effects	Yes
City fixed effects	Yes
*N*	3,976
Adj R-squared	0.9825

*T*-statistics based on robust standard errors are reported in parentheses. ***, **, and * indicate significance at the 1, 5, and 10% levels, respectively.

## 5 Discussion

### 5.1 Core findings and theoretical implications

Using a panel dataset of 284 prefecture-level cities in China from 2010 to 2023, this study treats the culture-tourism integration policy as a quasi-natural experiment and empirically examines its impact on urban ecological resilience. The results show that culture-tourism integration policies significantly improve urban ecological resilience, and this conclusion remains robust after a series of robustness checks and addressing endogeneity concerns. These findings carry important theoretical implications. First, this study broadens the research horizon of drivers of ecological resilience. Existing literature mostly focuses on natural endowment factors such as climate fluctuation and topography (7), or command-and-control policy instruments such as low-carbon city pilots and sponge city construction (8). In contrast, this study verifies that synergistic industrial policies like culture-tourism integration, which combine economic incentives and spatial restructuring, also serve as critical drivers of ecological resilience, thus enriching the policy evaluation toolkit. Second, this study deepens the understanding of the “green dividend” of culture-tourism integration policies. Mechanism tests reveal that culture-tourism integration policies enhance ecological resilience through three channels: industrial structure upgrading, green technology innovation, and public environmental awareness. This breaks the conventional focus on economic dimensions alone and uncovers the internal logic whereby industrial policies drive ecosystem restoration via the synergistic linkage of “economic structural optimization—technological support enhancement—social co-governance participation,” providing micro-level evidence for understanding the restructuring of human-environment interactions. Third, this study enhances the theoretical rigor by incorporating nonlinear characteristics and boundary conditions into the analytical framework. Previous studies often simplify the policy effects as a monotonic linear relationship, while this study explicitly recognizes that the impacts of culture-tourism integration policies are context-dependent and may exhibit heterogeneous or threshold patterns constrained by economic development level, resource endowments, and urban administrative hierarchy. The heterogeneous results further confirm that the policy effects vary substantially across regions and city types, which helps capture the contextual boundaries and nonlinear logic of ecological resilience governance. In this way, this study moves beyond a simplistic linear interpretation and provides a more realistic and nuanced theoretical understanding of how industrial policies shape urban ecological resilience.

### 5.2 Comparison with existing literature

The findings of this study both echo and substantially extend existing research. Regarding policy effects, the literature on the ecological impacts of tourism development presents a dual debate between ecological destruction and ecological conservation (5, 53). Some studies argue that tourism development may lead to overconsumption and environmental degradation. Based on causal inference using the multi-period difference-in-differences model, this study strongly supports the ecological conservation view, confirming that under sound policy guidance, culture-tourism integration can avoid development-induced damage and significantly improve ecological resilience. This conclusion refines earlier assessments based on static coupling coordination methods and identifies the net policy effect more precisely by addressing endogeneity (6). Regarding transmission mechanisms, most existing studies emphasize single mediating channels such as industrial structure or technological innovation. This study further introduces public environmental awareness as a social cognitive dimension, validating the role of culture-tourism integration policies as platforms for environmental education—namely, their ability to awaken public environmental awareness through implicit ecological value infiltration (33). This enriches the social interaction theory of environmental governance, indicating that culture-tourism integration is not merely an economic activity but also an important process for promoting social participation and collaborative governance. Regarding heterogeneity, this study finds stronger policy effects in non-designated historical and cultural cities. This counterintuitive result reveals the dialectical relationship between improvement space and constraint conditions. Unlike conventional studies emphasizing resource endowments as determinants of policy effectiveness, this study finds that historical and cultural cities, constrained by stricter protection rules and environmental capacity limits, partially restrict the release of policy dividends. In contrast, non-designated historical and cultural cities achieve faster improvements in ecological resilience due to latecomer advantages and larger room for enhancement. This provides a new academic perspective for reinterpreting the moderating role of resource endowments in policy outcomes.

### 5.3 Practical implications and alignment with SDGs

The conclusions of this study have clear policy implications and are highly consistent with SDGs. First, culture-tourism integration policies represent an effective pathway to SDG 3 (Good health and wellbeing) and SDG 11 (Make cities and human settlements inclusive, safe, resilient and sustainable). The results show that such policies significantly strengthen the resistance and recovery capacity of urban ecosystems, which may have potential implications for environmental exposure risks, living environmental quality, and public health co-benefits. These policies provide an important buffer against climate change and external shocks. Policymakers should incorporate ecological resilience and environmental health governance into core objectives of cultural and tourism development, strictly observe ecological protection redlines, eliminate extensive development patterns, and promote the transition from passive governance to proactive adaptation. Second, culture-tourism integration policies facilitate the synergy between SDG 8 (Decent work and economic growth) and SDG 12 (Responsible consumption and production). Mechanism analysis indicates that culture-tourism integration drives the green transformation of the economic system through industrial upgrading and green innovation. This green transformation may have potential implications for pollutant emissions and environmental health risks, potentially generating public health co-benefits. This suggests that local governments should guide factors to cluster in green and low-carbon industries via culture-tourism integration policies, stimulate enterprises’ endogenous motivation to adopt clean technologies by setting green standards and increasing innovation subsidies, and achieve win-win outcomes for economic development, ecological protection, and public health improvement. Third, heterogeneity analysis provides differentiated strategies for emerging economies to implement SDGs. For less developed regions and cities with relatively insufficient resources, culture-tourism integration can leverage latecomer advantages to rapidly improve ecological foundations by strengthening green infrastructure, which directly promotes environmental health equity. For developed regions and high-tier cities, policies should focus on innovative digital and intelligent ecological governance, which supports long-term public health security and high-quality urban development. In summary, by synergizing economic growth, green transformation, and spatial equity, enhancing urban ecological resilience through culture-tourism integration fundamentally consolidates the bottom line of public health. This demonstrates that locally tailored synergistic industrial policies are key to resolving the paradox between protection and development, offering replicable and scalable Chinese solutions for global environmental health governance and the comprehensive realization of SDGs.

## 6 Conclusions and policy recommendations

### 6.1 Conclusion

The main conclusions are as follows:

First, culture-tourism integration policies can significantly improve urban ecological resilience, and this conclusion remains valid after conducting a series of robustness checks and addressing endogeneity. Baseline regressions confirm that the implementation of culture-tourism integration policies significantly enhances urban ecological resilience. After addressing endogeneity via PSM-DID, placebo tests, the instrumental variable approaches, and double machine learning, the core conclusion remains plausible and consistent across a series of robustness tests, indicating that culture-tourism integration policies may serve as a useful tool for urban ecological governance.

Second, industrial structure upgrading, green technological innovation, and public environmental awareness are the key transmission mechanisms for culture-tourism integration policies to enhance urban ecological resilience. Mechanism tests demonstrate that such policies reduce production-side ecological pressure by promoting the rational and advanced transformation of the industrial structure; stimulate green technological innovation and improve environmental governance efficiency by establishing an incentive-compatible institutional environment; and enhance public environmental cognition and consolidate the social foundation of ecological governance by acting as a “platform for environmental education.” These three channels jointly drive urban ecological resilience from the dimensions of economic structure, technological support, and social cognition.

Third, the enhancement effect of culture-tourism integration policies on ecological resilience presents significant heterogeneity across geographical locations, resource endowments, and urban hierarchy. Geographically, the policy effect follows the order of West > Central > East, with stronger marginal effects in central and western regions due to greater ecological value transformation potential and latecomer advantages. In terms of resource endowments, the policy effect is significantly stronger in non-designated national historical and cultural cities than in national historical and cultural cities, as the latter are constrained by strict heritage protection and environmental capacity limits. In terms of urban hierarchy, municipalities directly under the central government and provincial capitals achieve significantly stronger policy effects than ordinary prefecture-level cities owing to their advantages in resource agglomeration and policy implementation.

### 6.2 Policy recommendations

Based on the above conclusions, the following policy recommendations are proposed to better leverage the role of culture-tourism integration policies in enhancing urban ecological resilience:

First, strengthen the ecological orientation of culture-tourism integration and uphold the philosophy of “shaping tourism with culture, highlighting culture through tourism, and prioritizing ecology.” Given the significant positive effect on ecological resilience, governments at all levels should abandon the extensive development model that solely pursues economic gains, and incorporate ecological resilience enhancement into the core objectives of cultural and tourism development. Ecological protection redlines must be strictly observed in policy and planning, with green and low-carbon principles embedded in the whole process of project development, facility construction, and operation. Institutional design should be used to amplify the positive ecological externalities of the cultural and tourism industry and prevent development-induced damage at the source.

Second, unblock policy transmission channels and build a collaborative governance system of “structural optimization—technological empowerment—social co-governance.” Promote industrial upgrading via culture-tourism integration, directing capital and technology to low-energy-consumption, high-value-added modern cultural and tourism services to reduce ecological pressure. Establish special funds for green technological R&D in the cultural and tourism sector, support demonstration projects such as smart culture-tourism and low-carbon scenic areas, and improve ecosystem risk monitoring and responses through digitalization. Utilize the implicit educational function of cultural and tourism scenarios, develop ecological research and nature experience products to enhance public environmental awareness, and form a multi-stakeholder governance pattern led by the government, implemented by enterprises, and supported by public participation.

Third, implement differentiated policies tailored to local conditions to address regional imbalances in culture-tourism development. For eastern regions with diminishing marginal effects, encourage the exploration of digital and intelligent ecological governance models to deepen the value of culture-tourism integration. For central and western regions, increase central fiscal transfer payments and infrastructure investment to transform ecological resource advantages into industrial advantages. For national historical and cultural cities, adopt a “micro-renovation and delicate upgrading” model to balance heritage protection and development. For non-designated national historical and cultural cities, build high-standard eco-friendly cultural and tourism facilities based on latecomer advantages. In addition, establish support mechanisms for underdeveloped regions and low-tier cities, compensate for fiscal and administrative shortcomings through targeted policy support and resource sharing, and enhance the inclusiveness and coordination of culture-tourism integration policies.

### 6.3 Research limitations and future directions

This study adopts a binary dummy variable to measure culture-tourism integration policies, which is applicable for identifying the average policy effect in the multi-period difference-in-differences framework but ignores differences in policy intensity and types across regions. Future research could construct continuous policy intensity indicators based on detailed text analysis of policy documents and classify diverse policy subtypes (e.g., tourism pilots, demonstration zones, cultural consumption initiatives) to capture their differentiated ecological impacts, thereby enriching the empirical evidence on policy design and implementation. Furthermore, this study employs interaction terms to capture continuous nonlinear effects within the difference-in-differences framework; future research could explore formal threshold models with appropriate data designs to identify specific structural breakpoints.

Additionally, from a methodological standpoint, several identification trade-offs warrant discussion. (1) Our core identification relies on the multi-period two-way fixed effects DID estimator. While robustness checks and the stable dynamic trend mitigate concerns, future research could formally apply heterogeneous treatment effect estimators to precisely decompose the average treatment effect across different adoption cohorts. (2) The instrumental variable strategy relies on the strong exclusion restriction that topography only affects ecological resilience via tourism policies. Future studies with more exogenous policy shocks or natural experiments are needed to provide definitive causal identification. (3) The mediation analysis identifies the directional existence of transmission channels but cannot fully capture the potential complex feedback loops among industrial upgrading, technological innovation, and social awareness. Structural equation modeling or dynamic network analysis might offer deeper insights into these synergistic mechanisms.

## Data Availability

The original contributions presented in the study are included in the article/Supplementary material, further inquiries can be directed to the corresponding author.
