# Supplementary material for: Does culture-tourism integration policy enhance urban ecological resilience? Evidence from a multi-period difference-in-differences analysis of Chinese cities
Source: Front Public Health. 2026 Jul 28;14:1870242. doi: 10.3389/fpubh.2026.1870242 (PMC13457463; doi:10.3389/fpubh.2026.1870242)
Supplement: Supplementary file 1 [file Data_Sheet_1.PDF]

TABLE A1 Balancing test for the instrumental variable.

**Variables**	**IV**
Lagged PGDP	0.0012 (1.05)
Lagged PD	-0.0008 (-0.64)
Lagged URBAN	0.0005 (0.38)
Lagged OPEN	-0.0014 (-1.12)
Lagged ERI	0.0009 (0.72)
Lagged FOREST	0.0011 (0.89)
Lagged ER	-0.0006 (-0.45)
Constant	0.0512** (2.15)
Year fixed effects	Yes
City fixed effects	Yes
N	3692
R-squared	0.0125

Notes: T-statistics based on robust standard errors are reported in parentheses. ***, **, and * indicate significance at the 1%, 5%, and 10% levels, respectively. The dependent variable is the instrumental variable (topographic slope difference×lagged national tourism revenue growth rate). The independent variables are one-period lagged city characteristics.

TABLE A2 Pre-policy placebo test for the instrumental variable (2005–2009).

Variables	First-stage (1) DID	Second-stage (2) ER
Instrumental variable	0.0006** (2.24)	
DID		0.0815 (0.82)
PGDP	0.0215** (2.18)	0.1205*** (4.52)
PD	0.0312 (1.42)	0.0854*** (3.65)
URBAN	0.0125 (1.15)	0.0058 (0.45)
OPEN	0.5520 (1.45)	0.6240 (1.28)
ERI	-0.1050 (-0.62)	-0.0850 (-0.44)
FOREST	0.0210 (1.25)	0.0325** (2.16)
Constant	-0.2050 (-1.48)	0.2850*** (3.85)
Kleibergen-Paap rk LM statistic	5.12 [0.0245]	
Kleibergen-Paap Wald rk F statistic	4.82 {16.38}	
Control variables	Yes	Yes
City/Year fixed effects	Yes	Yes
N	1280	1280
R-squared	0.865	

Notes: T-statistics based on robust standard errors are reported in parentheses. Values in [] are p-values; values in {} are the Stock-Yogo critical values at the 10% level. The sample period is restricted to 2005–2009, prior to the systematic implementation of culture-tourism integration policies. ***, **, and * indicate significance at the 1%, 5%, and 10% levels, respectively.

TABLE A3 Bootstrap test for mediation effects.

**Mechanism Path**	**Effect Type**	**Coefficient**	**95% CI (Lower)**	**95% CI (Upper)**	**Proportion Mediated**
DID → IS → ER	Indirect Effect	0.0012	0.0005	0.0020	24.0%
	Direct Effect	0.0038	0.0021	0.0056	
	Total Effect	0.0050	0.0035	0.0066	
DID → TECH → ER	Indirect Effect	0.0016	0.0008	0.0025	32.0%
	Direct Effect	0.0034	0.0018	0.0052	
	Total Effect	0.0050	0.0036	0.0065	
DID → PEA → ER	Indirect Effect	0.0008	0.0002	0.0015	16.0%
	Direct Effect	0.0042	0.0025	0.0060	
	Total Effect	0.0050	0.0034	0.0068	

*Notes: Bootstrap confidence intervals are based on 1,000 replications clustered at the city level.
